# Trait Plasticity among Invasive Populations of the Ant *Technomyrmex brunneus* in Japan

**DOI:** 10.3390/ani11092702

**Published:** 2021-09-15

**Authors:** Diyona Putri, Masanori Yokozawa, Toshiro Yamanaka, Adam L. Cronin

**Affiliations:** 1 Department of Biology, Graduate School of Science, Tokyo Metropolitan University, 1-1 Minami-Osawa, Hachioji, Tokyo 192-0397, Japan; 64.hopper.4.diab@gmail.com (M.Y.); adamcronin@gmail.com (A.L.C.); 2 School of Marine Resource and Environment, Tokyo University of Marine Science and Technology, 4-5-7 Konan, Minato-ku, Tokyo 108-8477, Japan; t.yamanaka@kaiyodai.ac.jp

**Keywords:** Formicidae, island, supercolony, social insects, genetic diversity

## Abstract

**Simple Summary:**

Invasive ants are a global economic and ecological problem. Understanding what makes them tick is thus an active area of scientific research. Many invasive ant species form large networks of inter-connected colonies (‘supercolonies’) which can span many hundreds of kilometres. Supercolonies are typically a characteristic of invasive populations, and studies have also identified shifts in other traits including diet in invasive populations compared to populations in the native range. This suggests that the ability to plastically change behaviour in this way may facilitate invasiveness. In this study, we assess trait plasticity in the invasive range of the ant *Technomyrmex brunneus*. This species is native to southern Asia and has invaded many islands of the southern Japanese archipelago, allowing us to compare traits among different invasive populations. We find a supercolony in one of the three invasive populations studied, though interestingly, this population did not show the expected pattern of lower genetic diversity. The supercolony population did differ in diet compared to other populations. While it is unclear if variation in these traits is linked, our findings add weight to arguments that plasticity is important in facilitating invasiveness.

**Abstract:**

Characters in invasive populations often differ from those in the native range, and the ability to express different characters may enhance invasive potential. Ants are among the most pervasive and damaging invasive species, by virtue of their transportability and broad-ranging ecological interactions. Their success is often attributed to the ability to exhibit different characteristics in invasive populations, including the formation of large, unicolonial associations (‘supercolonies’). It remains unclear, however, if such characteristics are a product or cause of the ecological dominance of invasive ants, and the advancement of our understanding has likely been restrained by the fact that studies to date have focused on a few globally important species with well-established invasions. In this study, we take advantage of an ongoing invasion of the tramp ant *Technomyrmex brunneus* in Japan to assess trait plasticity in the invasive range of this species. We find evidence for plasticity in social structure among island populations, with a supercolony evident on one of the three islands studied. Interestingly, we found no evidence of lower genetic diversity in this population, though natural isotope data indicate it was operating at a lower trophic level than other populations. These findings add weight to arguments that invasive species may benefit from the capacity to adaptively mould themselves to new ecological contexts.

## 1. Introduction

Social insects are among the most successful animal invaders and dominate the list of the most invasive terrestrial arthropods [1,2,3]. Invasive ants, in particular, can have global or near-global distributions [4], and are some of the most consequential invaders because of their broad range of direct and indirect ecological effects [5,6,7]. Understanding what makes some ant species such effective invaders, and in particular identifying whether invasiveness is associated with specific traits, is important for predicting which species may become invasive and thus critical for managing this global threat. Phenotypic plasticity—the ability to produce alternative phenotypes from the same genotype in response to environmental context [8]—has often been proposed as a mechanism facilitating invasion in different taxa [9,10,11]. While the evidence for a role of plasticity in supporting invasions has been inconsistent in plants [12,13], there is support of this idea in various animals [14,15,16]. Phenotypic plasticity may be particularly important in social insects, because their social nature capacitates plasticity in various forms [17]. Introduced populations of many of the most damaging invasive ants differ in social structure, behaviour, and/or genetic structure from those in the native range [6,18]. In particular, the ability to form unicolonial associations (supercolonies) and adaptively shift diet may help give invaders the edge [6,19]. Various invasive ant species form supercolonies in their invasive range [20], whereas such associations are largely absent in the native range but see [21,22]. Supercolonies can comprise hundreds or even thousands of nests and extend over tens or hundreds of kilometres, with extensive intermixing of both queens and workers among the component nests [20,23,24]. This is likely to confer advantages in terms of resource exploitation and competition, thus facilitating ecological dominance [25,26]. Dietary differences have also been reported between native and invasive populations of invasive ants. For example, both the Argentine Ant *Linepithema humile* and the Red Imported Fire Ant *Solenopsis invicta* exhibit shifts to lower trophic positions in their invasive ranges [27,28]. Such shifts have been explained through increased use of resources from mutualistic honeydew-producing insects [27,28]. Mutualisms such as this are found in various invasive ant species and can significantly boost growth and other performance characteristics [28,29,30,31].

Studies on trait plasticity in invasive species have to date have largely focused on well-established populations of major pest species, such as *L. humile* and *S. invicta* e.g., [24,32,33] and typically compare characteristics between native and invasive ranges. Correspondingly, we have a relatively poor understanding of the characteristics of more cryptic invaders and a relative paucity of information on variation among populations within the invasive range, though see [27,34,35]. This information is crucial for effective management of invasive species, as it can facilitate the identification of potential threats before they become established and help develop our understanding of adaptive shifts which may occur during the invasion process.

In this study, we take advantage of the ongoing invasion of *Technomyrmex brunneus* Forel, 1895 (*Hymenoptera*: *Formicidae*: *Dolichoderinae*) in the southern islands of Japan, to investigate trait plasticity among invasive populations of this species. This species is endemic to east, southeast, and south Asia and invasive in Japan, Korea, Borneo and New Guinea [36]. In Japan, *T*. *brunneus* is present as an invader in the southern-most mainland island, Kyushu, the oceanic islands of Ogasawara, and most, if not all, of the island chain extending from Kyushu to Taiwan. Reports of this species in Japan extend as far back as the early 1900s (though it was often misidentified as *T. albipes*) when it was reported in Kyushu [37]. It was established in Okinawa by the 1980s [38] and in the isolated archipelago of Ogasawara by 2005 [39], while it became established in Hachijojima [40] more recently (sometime before 2011) [41]. The distribution of invasive island populations permits a unique comparison of the life-history traits of different invasive populations. In *T*. *brunneus*, polydomous (multiple nest) colonies can consist of millions of adults, and may include both ergatoid (wingless) queens and males, as well as the usual alate (winged) sexual forms [38,42]. This species nests both on the ground and in trees and is therefore likely to directly compete for spatial resources with various native ants, and can cause damage and disturbance to areas of human habitation, particularly where it reaches high densities [41]. It is also an exploiter of honeydew [43], the sugar-rich secretions of various sap-sucking insects, and may be able to exclude native species from utilising these resources. Interestingly, *T*. *brunneus* in at least one invasive population in Japan appears to exhibit supercoloniality [40], while this has not been reported for other invasive populations. In this study, we use a combination of behavioural analysis, dietary inference, population genetics, and morphological character assessment to investigate variation in traits among different invasive island populations in this model system and explore how trait plasticity might facilitate invasiveness in this species.

## 2. Materials and Methods

### 2.1. Focal Species and Sample Sites

**Figure 1:**
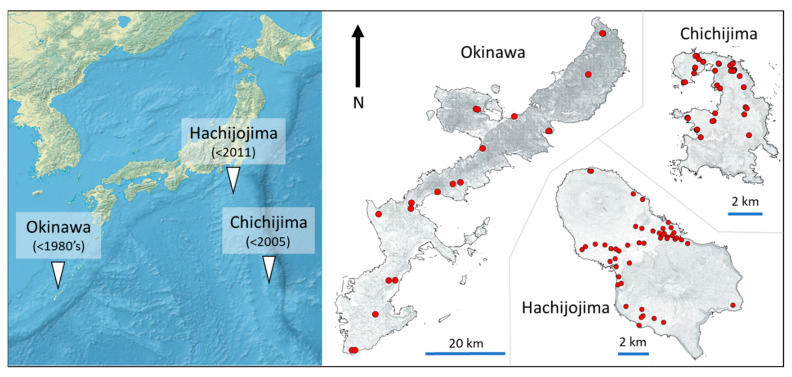
Sample sites of *T*. *brunneus* in this study. Earliest reported presence indicated in parentheses. Note different scale bars for each island in the right panel.

*Technomyrmex brunneus* ants were sampled from three island populations in the sub-tropical region south-east of the main islands of Japan between latitude 26 and 33° N (Figure 1): Hachijojima (47 sites) in September 2017, Okinawa (17 sites) in March 2018, and Chichijima (Ogasawara: 39 sites), in September 2018 (Figure 1 and Supplementary Material Table S1). Climate and land-use in the islands are broadly comparable, though the islands differ in size and degree of isolation, with Okinawa (1199 km^2^, 26°29’59″ N, 127°55’59″ E) by far the largest of the three, and Chichijima 23.45 km^2^, 27°07’44″ N, 142°21’78″ E) considerably more isolated than both Okinawa and Hachijojima (62.52 km^2^, 33°11’16″ N, 139°77’79″ E). These islands also differ considerably in invasion age: *T*. *brunneus* was established in Okinawa by the 1980s [38], in Ogasawara before 2005 [39,44], and relatively recently (by ~2011) in Hachijojima [40].

Ants were sampled using forceps from foraging trails or the upper parts of nests by removing the surface of logs of lifting rocks and transferred directly to 99% ethanol for genetic analysis or transferred to tubes with wet tissue for later behavioural observation. To assess how traits which might be associated with invasiveness vary among these populations, we assessed the following characteristics for ants in each location: supercolony status, trophic niche, population genetic structure, and morphological diversity. Details are provided for each of these factors below. In most cases, a random sub-sample of all colonies was used in each analysis, though samples from the four sites in north-eastern Okinawa were from traps and thus could not be used for isotope or behavioural analyses. Details on allocation of colonies to analyse are summarised in Table S1 of the Supplementary Material.

### 2.2. Assessment of Supercolony Status

Ants typically display high levels of aggression between colonies, and the absence of inter-colony aggression can be used as an indicator of supercoloniality [45]. Behavioural assays were conducted in a field laboratory the day the ants were collected. No workers were used in more than one assay. Two worker ants from different colonies were placed into a 5 cm Petri dish and allowed five minutes to interact. Petri-dishes were floored with paper disks which were replaced between tests. We randomly assigned pairs of individuals from different colonies for trials from the available pool of collected colonies in each population, using a total of 57 colonies (Hachijojima = 17, Okinawa = 12, Chichijima = 28). The behaviour of the ants was recorded using a webcam ELECOM UCAM-DLA200HBK and later scored from videos. Aggressive responses of ants were scored on a scale of 0–3, in which 0 = ignore (no antennation or aggression), 1 = interaction (contact with antennation), 2 = low aggression (attacking with opening of the mandibles but without biting), and 3 = high aggression (biting and pulling or fighting). We used two measures to estimate aggression level from these assays: the total number of interactions over the five-minute period and the mean aggression score of all interactions over the period, in both cases for both individuals in the pair. We could not perform tests of aggression between islands, as it was not possible to transport live specimens of an invasive species among locations.

### 2.3. Inference of Trophic Ecology

Natural isotope ratios of carbon (the ratio of ^13^C to ^12^C in parts per million, expressed as δ^13^C) and nitrogen (^15^N to ^14^N, expressed as δ^15^N) can be used to infer tropic niche, as the ratio of different isotopes of carbon can help discriminate among different sources of primary production (sugars), while that of nitrogen isotopes can be used to infer trophic position, because the δ^15^N of a consumer is typically enriched by ~3.0‰ for each trophic level [46,47,48]. As organismic isotope ratios can vary geographically [49,50], we assessed local baseline isotope ratios. In the absence of suitable plant species across all sites, we followed previous studies in using soil samples for this purpose [50,51]. Soil samples of ~10 cm^3^ were taken from the upper mineral soil layer (5–15 cm) within 1 m of each ant collection site and kept refrigerated until analysis. Ants were removed from alcohol within five days of the collection as extended storage in alcohol can influence isotopic measurements [46]. The gaster of ants was removed and the head and thorax used in the analysis. For nitrogen analysis, several ants were used from each site to comprise a sample between 0.5 and 1.3 mg (9–14 individuals) while a single ant (~0.05 mg) was used for carbon analysis. For soil samples we used 2.5 mg for nitrogen analysis and 0.5 mg for carbon. All samples were dried at 60 °C for 48 h then folded into tin capsules for analysis. Analysis of isotopic ratios was undertaken at Graduate School of Natural Science and Technology, Okayama University, Japan using a continuous-flow mass spectrometer coupled with an elemental analyser (IsoPrime EA; GV Instruments, Manchester, UK). All isotopic values are reported as common δ^15^N/δ^13^C notation, as per million deviations relative to international standards. The analytical error during the overall process of mass spectrometry was less than 0.2 ‰. Baseline corrected values of δ^15^N and δ^13^C were calculated for each sampling site as ant values—soil values.

### 2.4. Population Genetic Analysis

A single worker was taken from each colony for genetic analysis. DNA was extracted following the “Chelex-TE-ProK protocol” (for details see [52]) using the whole body of the ant. We obtained a whole-genome reduced-representation library using MIG-Seq (multiplexed ISSR genotyping by sequencing), a PCR-based method for genome-wide identification of single-nucleotide polymorphisms using next-generation sequencing [53]. This approach relies on MIG-Seq primers designed to anneal to repetitive motifs across the genome. The method generates fewer total loci than RAD-Seq but is applicable to lower quality DNA. Our MIG-Seq dataset comprised 79 individuals from three populations of *T*. *brunneus*. Data acquisition followed Suyama and Matsuki [53] except for the following: we used Trimmomatic to remove the reads derived from extremely short library entries and to remove the SSR region and anchor sequences from Read 2 sequences, and then used the analytical pipeline ipyrad v. 0.9.44 [54] to identify putative SNPs. We used the default parameter settings in ipyrad except for the value of clust_threshold, which we set at 0.95. This produced a final catalogue of 10,532 putative SNPs. We filtered these SNPs using vcftools version 0.1.14 [55], removing loci which were not present in at least 75% of individuals, and then filtered out samples in which less than 60% of loci were present, yielding a final dataset of 428 SNPs.

We used the R package *adegenet* [56] to quantify the following population genetic metrics: the number of alleles (*N_A_*), allelic richness (*A_R_*), expected heterozygosity (*H_E_*), observed heterozygosity (*H_O_*), and inbreeding coefficient (*F_IS_*). Isolation by distance was assessed using a Mantel test implemented in the *ade4* package of R [57]. Genetic variation between and within populations was assessed with AMOVA implemented in the *poppr* package of R [58]. We also determined inter-population Weir and Cockerham [59] *F_ST_* in *hierfstat* [60]. We assessed all loci for Hardy–Weinberg equilibrium within each population using the hw.test function from the *pegas* package of R [61], correcting for multiple testing using the Benjamini–Hochberg method [62]. For both *F_ST_* and *F_IS_*, we determined 95% confidence intervals (CIs) of estimates using 10,000 bootstraps and considered values significantly different to zero if CIs did not span zero. Finally, we used DAPC (discriminant analysis of principal components) implemented in *adegenet* to describe genetic clusters of individuals using K-means clustering of principal components. We ran K-means clustering with different numbers of clusters (K = 1–10) and identified the optimal number of clusters as the value of K with the lowest BIC.

### 2.5. Morphological Analysis

**Figure 2:**
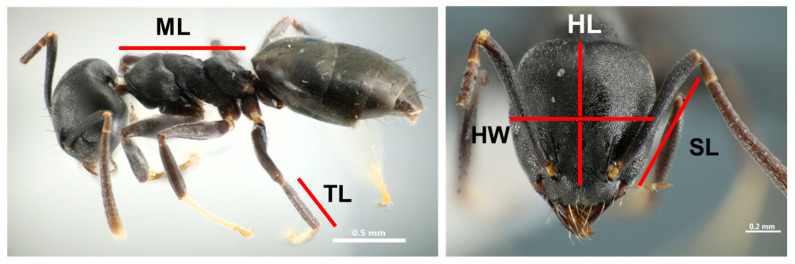
Five morphological characters measured for *T. brunneus* workers: Head Width (HW), Head Length (HL), Tibia Length (TL), Mesosoma Length (ML), and Scape Length (SL).

Founder effects can lead to change in morphological characters or reduced morphological diversity within colonies, which can limit the capacity for workers to specialise on different tasks (division of labour) and thus reduce colony efficiency, which relies on this diversity [63,64,65,66,67]. We assessed if different populations displayed differences in morphology and/or morphological diversity as follows. We measured dry, pinned specimens using ImageJ 1.47 (http://imageJ.nih.gov/ij/; accessed on 20 January 2020) based on photographs taken using a Canon EOS Kiss X9 digital camera attached to Nikon AZ100 stereomicroscope. We measured five morphological characters for five workers from each of 10 colonies from each population: Head Width (HW): maximum width of head including eyes, measured in full-face view; Head Length (HL): maximum length of head in full-face view; Tibia Length (TL): maximum length of the hind femur, measured in anterior view; Mesosoma Length (ML): diagonal length of the mesosoma in profile from the pronotum to the posterior basal angle of the propodium; Scape Length (SL): maximum length of antennal scape (Figure 2). These characters were used as they are general size indicators and likely to be associated with functional performance such as foraging. For each morphological trait in each colony from each population, we also calculated the coefficient of variation (COV) of five measured workers, as the ratio of the standard deviation to the mean, to provide a measure of intra-colony morphological diversity.

### 2.6. Statistical Analysis

Statistical analyses were performed in R v. 4.0.2 [68]. For aggression data, we used general linear models (GLM) to investigate the effects of island and distance between sites within each island on a) mean aggression score and b) number of interactions over the five-minute trials. The number of interactions was first log (x + 1) transformed to fit the assumptions of parametric models. The influence of different factors was determined using likelihood ratio tests from the R package *lmtest* [69] on models with and without the factor of interest. This was followed by post-hoc tests for pairwise comparisons between islands using the glht function from the *multcomp* package [70]. Isotope ratios for carbon and nitrogen data did not satisfy assumptions of parametric analysis so were compared using Kruskal–Wallis tests followed by Dunn post-hoc tests. The dataset of five morphological characters was subjected to a principal component analysis (PCA) using the R package *factoextra* [71]. PCA factors and measures of the COV for each morphological measure were then compared between islands using Kruskal–Wallace tests followed by Dunn post-hoc tests, as some PC axes and COV data did not fit assumptions for parametric analyses. Data were assessed for normality using Shapiro–Wilk’s tests. Significance values in multiple comparisons were adjusted following Benjamini and Hochberg [62].

## 3. Results

### 3.1. Assessment of Supercolony Status

**Figure 3:**
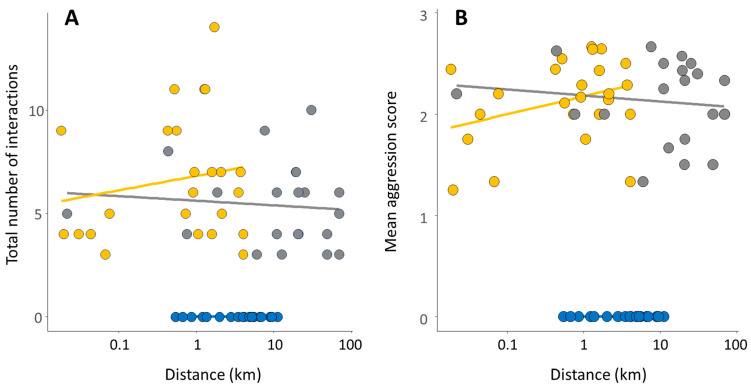
Scatterplot with regression lines for total number of interactions (**A**) and mean aggression scores (**B**) for five-minute pairwise trials within each population in relation to the geographical distance between test-pair colonies. A total of 67 trials were run from three populations (Hachijojima (blue circles, *n* = 22), Okinawa (grey circles, *n* = 21), and Chichijima (yellow circles, *n* = 24)).

We found clear differences in intra-colony aggression between populations. Aggressive behaviours were not observed in Hachijojima, with all assays obtaining a score of 0 (no aggression), while we found consistently high aggression, with scores of 2 or 3, in Okinawa and Chichijima (Figure 3). Both the number of interactions and the mean aggression score differed significantly between islands (LRT: number of interactions: χ^2^ = 161.09, *p* < 0.001; mean aggression score: χ^2^ = 158.01, *p* < 0.001). Pairwise comparisons indicated no difference between Okinawa and Chichijima for number of interactions or aggression score (z = −1.67, *p* = 0.21 and z = −0.55, *p* = 0.84, respectively), while Hachijojima differed significantly from both Okinawa and Chichijima for number of interactions (z = 19.88, *p* < 0.001, and z = −21.09, *p* < 0.001, respectively) and mean aggression score (z = 20.26, *p* < 0.001, and z = −19.95, *p* < 0.001, respectively). There was no influence of distance between colony pairs on aggression within islands overall (LRT: number of interactions: χ^2^ = 0.16, *p* = 0.69; mean aggression score: χ^2^ = 0.70, *p* = 0.41) and no interaction between distance and island (LRT: number of interactions: χ^2^ = 1.77, *p* = 0.41; mean aggression score: χ^2^ = 3.97, *p* = 0.14).

### 3.2. Inference of Trophic Niche

**Figure 4:**
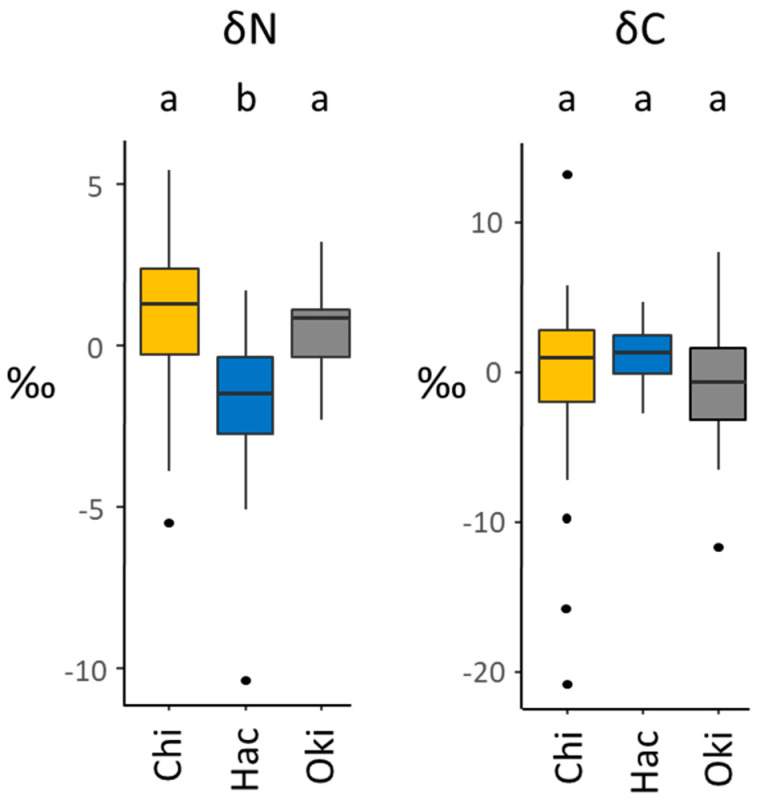
Boxplots of baseline-corrected natural isotope ratios for δ^15^N (**left**) and δ^13^C (**right**) for *T. brunneus* sampled from three island sites (Chi = Chichijima, Hac = Hachijojima, Oki = Okinawa) in parts-per-million. Letters above bars denote groups inferred by Dunn’s test. Boxes represent quartiles, the horizontal bar indicates the median, whiskers represent the minimum and maximum values, and points indicate outliers.

Baseline-corrected isotope values of ants varied significantly between islands for nitrogen (Kruskal–Wallis: χ^2^ = 23.59, df = 2, *p* < 0.001) but not for carbon (Kruskal–Wallis: χ^2^ = 2.93, df = 2, *p* = 0.23). Post-hoc tests indicated that δ^15^N values for Hachijojima were significantly lower than both Chichijima (z = 4.43, *p* < 0.001) and Okinawa (z = −3.21, *p* = 0.001), but that there was no difference between the latter two sites (z = 0.56, *p* = 0.287; Figure 4). At the same time, the range of values within each site was comparable for δ^15^N and spanned ~6–8‰ (excluding outliers), while Hachijojima exhibited a slightly lower range of values for δ^13^C than either Okinawa or Chichijima.

### 3.3. Population Genetic Analysis

The population genetic summary statistics reveal no marked differences in genetic diversity between sites (Table 1), and thus no indication of the expected pattern of lower genetic diversity associated with the supercolony in Hachijojima.

**Figure 5:**
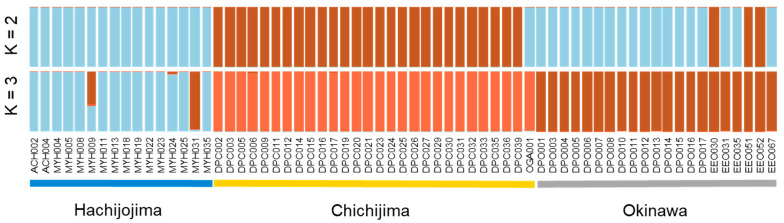
Compoplot from DAPC (discriminant analysis of principal components) for *T*. *brunneus* sampled from three island populations, for *K* = 2 (inferred most likely value) and *K* = 3.

We found no significant pattern of isolation by distance (IBD) at any site, though inbreeding coefficients were significantly different from zero at all sites, and all sites also exhibited deviations from HWE at some loci. Pairwise *F_ST_* values between islands indicated low but significant genetic variation between populations (Hachijojima–Okinawa: *F_ST_* = 0.042, 95% CIs = 0.021–0.065; Chichijima–Okinawa: *F_ST_* = 0.090, 95% CIs = 0.061–0.121; Hachijojima–Chichijima: *F_ST_* = 0.140, 95% CIs = 0.097–0.184). AMOVA indicated that 66.8% of the variation was within individuals, 23.8% within populations and 9.4% between populations, with this differentiation significant at all levels (*p* = 0.01 in each case). The DAPC analysis inferred that the optimal clustering was obtained with *K* = 2, and we summarise these results and those for *K* = 3 in Figure 5 (see also Supplementary Material Figure S1). Samples in the *K* = 2 analysis segregated largely into Chichijima and (Okinawa + Hachijojima) clusters. When considering a *K* of 3, the pattern of clustering indicated that Chichijima samples were separated from other populations, while admixture occurred between populations from Hachijojima and Okinawa. Overall, these data indicate that there was low genetic variation within all populations, and no indication that the supercolony population in Hachijojima was structured differently to other sites. Island populations were genetically distinct from each other, though there was a higher affinity between Okinawa and Hachijojima than either of these islands and Chichijima.

### 3.4. Morphological Analysis

**Figure 6:**
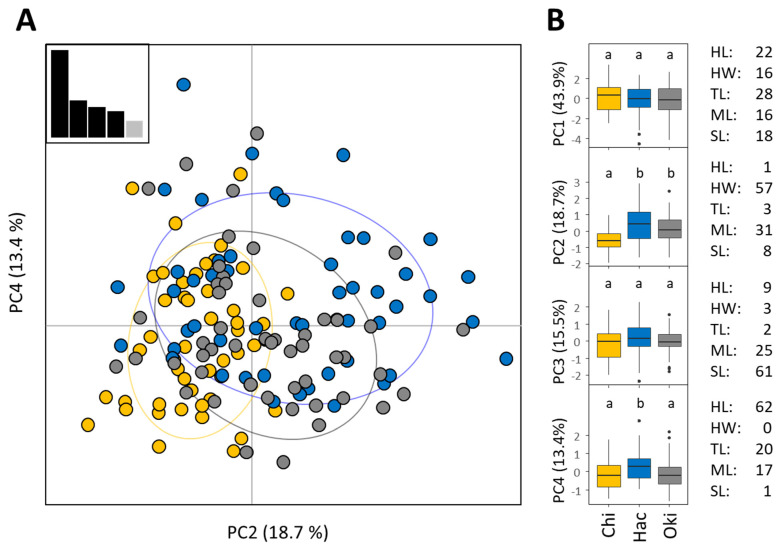
(**A**) PCA analysis based on five morphological characters of *T*. *brunneus* from three island populations. The PC axes 2 and 4 are shown as only these exhibited significant differences between populations. Yellow circles indicate ants from Chichijima, blue from Hachijojima and grey, Okinawa. Inset: plot of eigenvalues of PC axes. (**B**) Boxplots of four main PC axes and percentage contribution of each morphological character to that PC axis. Characters were HW (Head Width), HL (Head Length), TL (Tibia Length), ML (Mesosoma Length), and SL (Scape Length). In the boxplot figure, boxes represent quartiles, the horizontal bar indicates the median, whiskers represent the minimum and maximum values, and points indicate outliers. Letters above boxes show groups indicated by Dunn’s test. Chi = Chichijima, Hac = Hachijojima, and Oki = Okinawa.

We found only minor variation in morphological characters between populations (Figure 6). Comparisons among islands for each PC axis indicated two differences for minor axes: Chichijima was significantly lower than other islands for PC2 (explaining 19% of variation, principally HW) while Hachijojima was significantly higher for PC4 (explaining 13% of variation, principally HL). The degree of worker size diversity (COV) within colonies did not differ for any of the five characters (Supplementary Material Figure S2).

## 4. Discussion

In this study, we compare the life-history characteristics of three invasive populations of the ant *Technomyrmex brunneus* in the southern islands of Japan. Genetic analyses indicated that, while all populations were genetically distinct, the Hachijojima and Okinawa populations were genetically more closely associated with one another than either population was with the Chichijima population. This is consistent with the relative isolation of Chichijima, which is part of the Ogasawara archipelago, an isolated oceanic island chain ~1000 km from the Japanese mainland and ~1500 km from Okinawa. In contrast, both Hachijojima and Okinawa are of continental origin and connected by numerous other intermediate islands. Nonetheless, the differences in life-history characteristics we identified were not consistent with the genetic relationships between populations. Our results indicate the population in Hachijojima constitutes a supercolony, confirming earlier reports [40], while we found no evidence of supercoloniality in Chichijima or Okinawa. There was no pattern to indicate that this difference coincided with lower genetic diversity in the Hachijojima population, as population genetic metrics did not differ markedly between sites. Similarly, there was only limited evidence of variation in worker morphology between populations. On the other hand, our analysis of natural isotope ratios indicated that the Hachijojima population was using a lower trophic level than the other two populations.

Supercolonies can arise because of a breakdown of the recognition system ants use to discriminate ‘friends’ (nestmates) from ‘foes’ (non-nestmates) [6,33,72]. This recognition system is typically based on multicomponent cues encoded in cuticular hydrocarbon (CHC) profiles, with workers displaying aggression toward ants that possess different CHC profiles [73,74,75]. Studies comparing populations in the native range to those in the invasive range have reported patterns consistent with a causal influence of genetic bottlenecks such as founder effects, which could lead to reduced aggression by reducing the diversity of CHC profiles in invasive populations [20,33,45,76,77,78]. However, other studies have found a lack of evidence to support a genetic basis for reduced aggression in supercolonies [23,79]. Giraud et al. [23] proposed an alternative ‘genetic cleansing’ hypothesis, under which high nest densities achieved by invaders released from parasite and predation pressure leads to selection against aggression, because of the high costs imposed by constant fighting. This loss of aggression could occur without generalised loss of genetic diversity if changes are limited to loci related to nestmate recognition [23]. The genetic characteristics of invasive populations of *T. brunneus* in Japan seem typical of invasive ants in exhibiting low genetic diversity and a lack of genetic isolation by distance (IBD) [23,24,80]. That we found no evidence of lower genetic diversity in with the supercolony population in Hachijojima relative to other populations may suggest that this difference has an ecological basis, possibly lending support to the ‘genetic cleansing’ hypothesis. However, while this population exhibits very high nest densities [40,41] and is thus is likely to have high intra-specific competition, the very recent invasion here (thought to have occurred around 2011) leaves little time for the required evolutionary change to occur, arguing against this possibility.

The Hachijojima population was also characterised by a lower trophic level than either Okinawa or Chichijima populations. Shifts to lower trophic levels have been reported in the invasive range of *L. humile* [27] and *S. invicta* [28], while invasive populations of *Formica paralugubris* were both up-shifted and down-shifted compared to native range populations [34]. Trophic shifts in ants are typically associated with variation in the reliance on carbohydrates obtained from honeydew producing insects. It is thought that this shift is either preferential, and made possible because of limited competition for these resources from native ants (the Resource Preference Hypothesis), or through necessity, because of competition for other resources (the Resource Limitation Hypothesis [31]). Previous studies have shown that invasive species can exclude native species from carbohydrate resources (e.g., *S. invicta* [81]), possibly because of the boost invasive species can enjoy through release from parasite pressures [79,82,83]. It has been reported that carbohydrate resources from hemipteran mutualists can form the majority of the diet for *Technomyrmex* ants [43,84]. The lower trophic level at Hachijojima may thus reflect a preferential shift toward honeydew resources in this population and could explain the lower range of δ^13^C values observed in this population. This might also help explain the extremely high densities reported in this population, as carbohydrate supplementation can fuel colony growth, worker activity and aggression in ants, thus enhancing competitive performance [29,31,81]. Why Hachijojima appears unique in this regard is unclear, though this may be related to the strength of competition and/or age of invasion. Trophic level has been shown to vary temporally during invasion in Argentine ants [27], and the invasion in Hachijojima is more recent than in Chichijima (<2005) or Okinawa (<1980s). However, competition from other ant species is also likely to vary among islands, and in particular from other invasive species which heavily exploit honeydew such as *Anoplolepis gracilipes* (which is found only in Okinawa: [84]). Unfortunately, a lack comparable survey data of ants and resource use precludes a comparative analysis of competitive ant communities at this time. An additional possibility is a link between supercolony status and diet, which is theoretically possible because of the influence diet can have on CHC profiles [85,86]. An increased reliance on honeydew resources, or canalisation of trophic niches, could potentially erode important diet-related components from the CHC profile, leading to reduced aggression [27,29]. This possibility also awaits further research.

## 5. Conclusions

In this study, we assessed inter-population plasticity in traits likely to be associated with invasiveness in *T. brunneus* in Japan, by comparing social structure, trophic niche, population genetics, and morphology among three invasive populations. We find evidence of plasticity in trophic niche and social structure, with a supercolony and use of a lower trophic niche in Hachijojima. This supercolony was not associated with lower genetic diversity, suggesting an ecological basis for this difference. While it is possible that there were causal links between supercolony status and the differences in trophic level, we cannot rule out other influences such as differences in the timing of invasion and/or differing ant communities between sites. These data lend support to the idea that plasticity is common in invasive species and may be a facilitator of invasiveness [9,10,11]. While it remains unclear if traits observed in invasive ants are a product or cause of their ecological dominance [87,88], a success factor of invasive ants is likely to be the ability to adaptively mould themselves to suit novel ecological contexts. Studies of *T. brunneus* in the native range are needed to establish if the traits exhibited within invasive populations in Japan are pre-adaptive or the result of ongoing evolution during the invasion process [14,15,16,87,89]. The potential for variation in other life-history traits of this species also deserves further scrutiny, as factors including the degree of polydomy, polygyny, and the presence of wingless, ergatoid queens [38,42] may vary among populations. Further studies of *T. brunneus* and other ongoing invasions will help develop our understanding of the degree to which dietary shifts and supercoloniality can independently or collectively facilitate invasiveness in ants.

## Figures and Tables

**Table 1:** Descriptive statistics of genetic diversity of *T*. *brunneus* for each population, generated from 428 SNPs. The number of colonies tested (one worker per colony) is indicated by N. Other statistics are described in Section 2.

Population	N	The Number of Alleles (*N*_A_)	Allelic Richness (*A_R_*)	Observed Heterozygosity (*H*_O_)	Expected Heterozygosity (*H_E_*)	Inbreeding Coefficient (*F_IS_*) (95% CIs)	Loci in HWE (%)	Isolation by Distance (IBD) *p*-Value
Hachijojima	16	580	1.22	0.061	0.084	0.271 (0.197–0.345)	97.4	0.986
Okinawa	21	675	1.50	0.055	0.088	0.372 (0.313–0.431)	90.0	0.058
Chichijima	28	615	1.37	0.048	0.063	0.228 (0.173–0.281)	98.8	0.156

## Data Availability

All data used in this study are provided as supplementary material to this article.

## Supplementary Materials

The following are available online at https://www.mdpi.com/article/10.3390/ani11092702/s1, Table S1. Sampling sites and breakdown of analytical methods for *T*. *brunneus* samples. Figure S1. DAPC scatterplot for all populations. Figure S2. Boxplot of intra-colony coefficient of variation (COV) for each morphological trait between islands.
