# Supplementary material for: Trait Plasticity among Invasive Populations of the Ant Technomyrmex brunneus in Japan
Source: Animals (Basel). 2021 Sep 15;11(9):2702. doi: 10.3390/ani11092702 (PMC8465827; doi:10.3390/ani11092702)
Supplement: Supplementary file 1 [file animals-11-02702-s001.zip › animals-1340838-supplementary.pdf]

Table S1. Sampling sites and breakdown of analytical methods for *T. brunneus* samples. Geographic coordinates are given in decimal degrees. Usage of each sample for different analyses indicated by circles (see main methods).

Island	Sample ID	Latitude	Longitude	Aggression assays	Isotopic analysis	Population genetic analysis	Morphological analysis
Hachijojima	MYH001	33.1204	139.8087		o		
	MYH002	33.1196	139.8086		o		
	MYH003	33.1197	139.8074		o		o
	MYH004	33.1133	139.7874		o	o	o
	MYH005	33.1157	139.7924		o	o	
	MYH006	33.1151	139.7950		o		
	MYH007	33.1212	139.8062		o		
	MYH008	33.1572	139.7596	o	o	o	
	MYH009	33.1571	139.7583		o	o	o
	MYH010	33.1448	139.7857	o	o		
	MYH011	33.1416	139.7915	o	o	o	o
	MYH012	33.1091	139.7802		o		
	MYH013	33.1095	139.7779	o	o	o	
	MYH014	33.1100	139.7754		o		
	MYH015	33.1119	139.7714	o	o		o
	MYH016	33.1109	139.7650	o	o		
	MYH017	33.1105	139.7594		o		
	MYH018	33.1077	139.7567		o	o	
	MYH019	33.1022	139.7752	o	o	o	
	MYH020	33.1035	139.7775		o		
	MYH021	33.1190	139.8137	o	o		
	MYH022	33.1187	139.8176	o	o	o	
	MYH023	33.1170	139.8221		o	o	
	MYH024	33.0809	139.8526	o	o	o	o
	MYH025	33.0662	139.8110	o	o	o	o
	MYH026	33.0689	139.8045		o		
	MYH027	33.0700	139.7982		o		
	MYH028	33.0640	139.7955	o	o		
	MYH029	33.0693	139.7968		o		
	MYH030	33.0736	139.7972	o	o		
	MYH031	33.0753	139.7866	o	o	o	o
	MYH032	33.0934	139.7809	o	o		
	MYH033	33.0877	139.7814		o		
	MYH034	33.0891	139.7825		o		
	MYH035	33.0992	139.7793	o	o	o	
	MYH036	33.1020	139.7869		o		
	MYH037	33.1210	139.8116		o		

	MYH038	33.1197	139.8151		o		
	MYH039	33.1230	139.8131		o		
	MYH040	33.1256	139.8114		o		
	MYH041	33.1284	139.8085		o		
	MYH042	33.1249	139.8060		o		
	MYH043	33.1224	139.8035		o		
	ACH001	33.1222	139.8073		o		
	ACH002	33.1232	139.8000		o	o	o
	ACH003	33.1235	139.7932		o		
	ACH004	33.1250	139.7887		o	o	o
Okinawa	DPO001	26.0869	127.6781	o	o	o	o
	DPO003	26.0867	127.6856	o	o	o	o
	DPO004	26.2523	127.7799	o	o	o	o
	DPO005	26.4082	127.7411			o	
	DPO006	26.6571	127.9717	o	o	o	o
	DPO007	26.6562	127.9759	o	o	o	
	DPO008	26.5634	127.9864		o	o	o
	DPO010	26.4345	127.8188	o	o	o	o
	DPO011	26.4347	127.8188	o	o	o	
	DPO012	26.4795	127.9164	o	o	o	o
	DPO013	26.4833	127.9344	o	o	o	o
	DPO014	26.4606	127.8802	o	o	o	o
	DPO015	26.4213	127.8173	o	o	o	
	DPO016	26.2518	127.7646	o	o	o	
	DPO017	26.1718	127.7332			o	o
	EEO030	26.8360	128.2719			o	
	EEO031	26.8363	128.2705			o	
	EEO035	26.7389	128.2372			o	
	EEO051	26.6045	128.1426			o	
	EEO052	26.6046	128.1435			o	
	EEO067	26.6397	128.0626			o	
Chichijima	DPC001	27.0642	142.1943	o			
	DPC002	27.0642	142.1946	o		o	
	DPC003	27.0644	142.1947	o	o	o	o
	DPC004	27.0690	142.2034	o			
	DPC005	27.0689	142.2028	o	o	o	
	DPC006	27.1003	142.1979	o	o	o	
	DPC007	27.1002	142.1978	o			
	DPC008	27.0969	142.1934	o			
	DPC009	27.0973	142.1936	o	o	o	
	DPC010	27.1034	142.1945	o			
	DPC011	27.1033	142.1937	o	o	o	
	DPC012	27.1016	142.1955	o	o		o

DPC013	27.0992	142.2061				
DPC014	27.0994	142.2060	o	o	o	
DPC015	27.0983	142.2122	o	o	o	
DPC016	27.0994	142.2135	o		o	
DPC017	27.0960	142.2144	o	o	o	
DPC018	27.0955	142.2142	o			
DPC019	27.0955	142.2140	o	o	o	o
DPC020	27.0954	142.2125	o	o	o	
DPC021	27.0927	142.2172	o	o	o	
DPC022	27.0868	142.2194	o			
DPC023	27.0724	142.2196	o	o	o	o
DPC024	27.0762	142.2204	o	o	o	
DPC025	27.0756	142.2210	o	o	o	o
DPC026	27.0614	142.2222	o	o	o	
DPC027	27.0642	142.1943	o	o	o	o
DPC028	NA	NA	o			
DPC029	27.0942	142.1930		o	o	o
DPC030	27.0894	142.1875	o		o	
DPC031	27.0895	142.1882		o	o	o
DPC032	27.0956	142.2041		o	o	o
DPC033	27.0878	142.2053		o	o	
DPC034	27.0730	142.2042	o			
DPC035	27.0603	142.1965	o	o	o	
DPC036	27.0707	142.1896	o	o	o	o
DPC037	27.0703	142.1897		o		
DPC038	27.0861	142.2068	o			
DPC039	27.0942	142.1930				
OGA001	27.0615	142.2218			o	

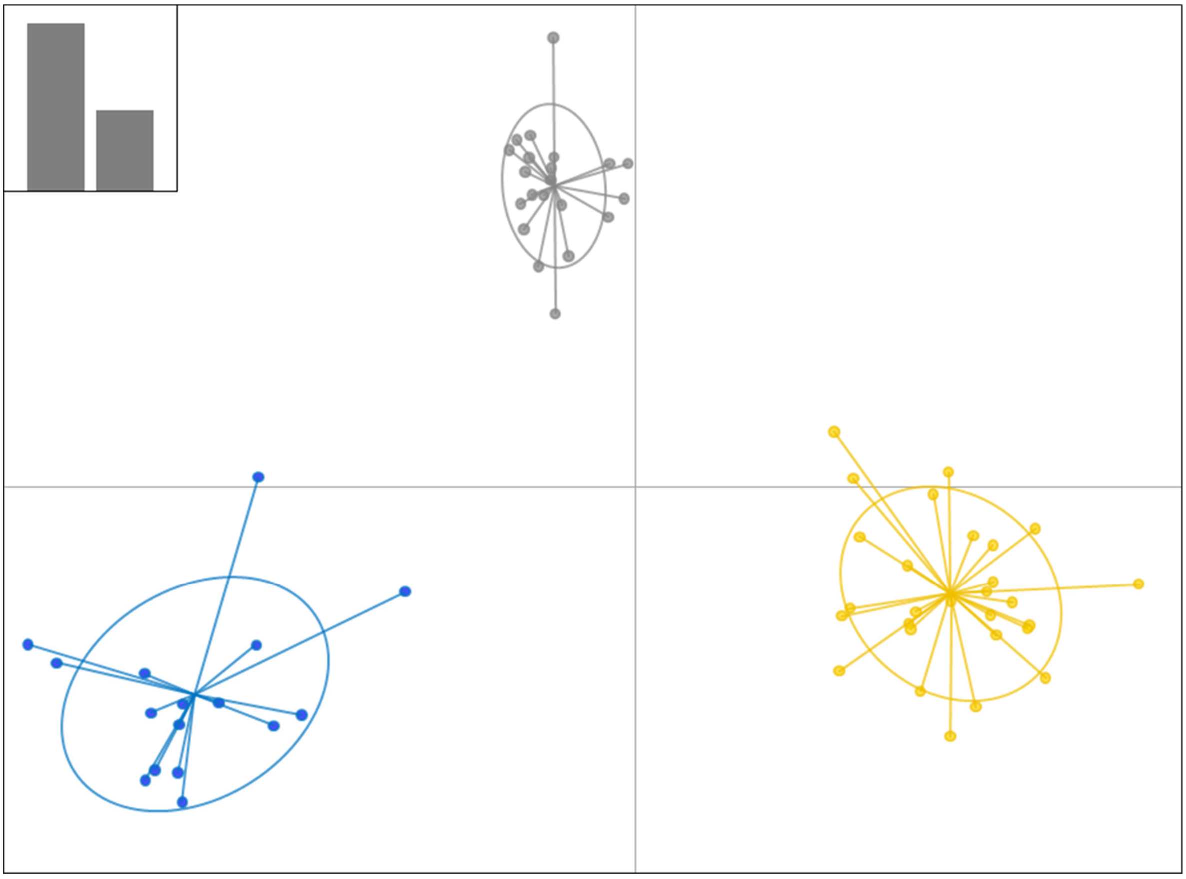

Figure S1. DAPC scatter plot of three populations based on 428 SNPs. Inset shows eigen values for two main discriminate analysis axes. Gold points indicate Ogasawara, blue Hachijojima, and grey Okinawa.

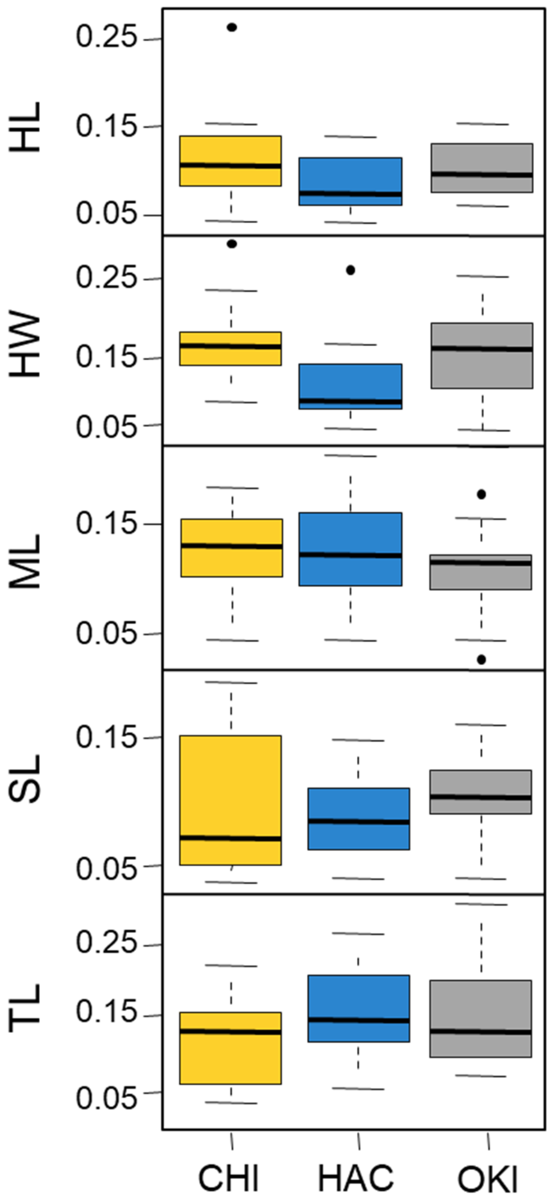

Figure S2. Box plot of intra-colony coefficient of variation (COV) for each morphological trait between islands. There was no significant difference for any of the five morphological characteristics (ANOVA: HW (F_2 = 1.86, P = 0.175), HL (F_2 = 1.626, P = 0.215), TL (F_2= 0.909, P = 0.415), ML (F_2 = 0.506, P = 0.608) and SL (F_2 = 0.301, P = 0.734)). Gold bars indicate Ogasawara, blue Hachijojima, and grey Okinawa.
